# Salvage Management of Anterior-Posterior Compression (APC) Type II Pelvic Ring Injury Using Isolated Posterior Fixation After Anterior Implant Removal: A Case Report

**DOI:** 10.7759/cureus.116481

**Published:** 2026-09-18

**Authors:** Sireethorn Chatpermporn, Pamok Saengsirinavin

**Affiliations:** 1 Orthopedics, Police General Hospital, Bangkok, THA

**Keywords:** fractures of the pelvis and acetabulum, minimally invasive pelvic ring fixation, ortho, pelvic ring injury, trauma injuries

## Abstract

Anterior-posterior compression (APC) injuries of the pelvic ring type II have been classically managed with anterior fixation, with or without posterior fixation. However, anterior approaches may be associated with wound complications, particularly in the setting of urologic or soft tissue injury. The role of isolated posterior fixation in this context remains unclear.

We report a case of a 30-year-old male with an APC type II pelvic ring injury initially treated with combined anterior plating and posterior iliosacral screw fixation. The postoperative course was complicated by a deep surgical site infection at the anterior incision, necessitating implant removal and serial debridement. The posterior fixation was retained. Radiographic follow-up demonstrated maintenance of pelvic alignment, with anterior symphyseal diastasis stabilizing at 8 mm. Clinically, the patient achieved pain-free full weight-bearing by three months and returned to unrestricted ambulation at 11 weeks, with no evidence of instability.

This case demonstrates that isolated posterior fixation may provide sufficient stability to allow functional recovery in selected APC type II injuries following anterior implant removal. This approach should be considered a salvage strategy rather than a primary treatment option. Further studies are required to define its indications.

## Introduction

Anterior-posterior compression (APC) injuries of the pelvic ring represent a spectrum of ligamentous disruption progressing from anterior to posterior structures. APC type II injuries are characterized by disruption of the pubic symphysis and anterior sacroiliac ligaments, resulting in rotational instability while maintaining vertical stability [1-4].

The optimal surgical management of APC type II injuries remains debated [4,5]. While isolated anterior fixation has traditionally been considered sufficient, increasing evidence supports the addition of posterior fixation to improve stability and reduce implant failure [6,7]. However, anterior approaches, particularly via a Pfannenstiel incision, may be associated with wound complications, especially in patients with concomitant urologic injuries [8-10].

The role of isolated posterior fixation in APC type II injuries is not well established. We present a case in which anterior fixation was removed due to a deep infection, leaving posterior fixation as the sole stabilizing construct, with favorable clinical and radiographic outcomes.

## Case presentation

A 30-year-old male presented following a high-energy motorcycle collision. On arrival, he was hemodynamically stable (blood pressure 119/82 mmHg, pulse 80 bpm) with a Glasgow Coma Scale score of 15. Physical examination revealed tenderness over the pubic region. Focused assessment with sonography for trauma (FAST) was negative.

Initial pelvic radiographs demonstrated a 12 mm diastasis of the pubic symphysis and 2 mm widening of the left sacroiliac joint compared with the contralateral side. After application and adjustment of a pelvic binder, repeat imaging showed reduction of symphyseal diastasis to 7.5 mm. Computed tomography confirmed an APC type II pelvic ring injury with mild sacroiliac joint widening (Figure 1).

**Figure 1:**
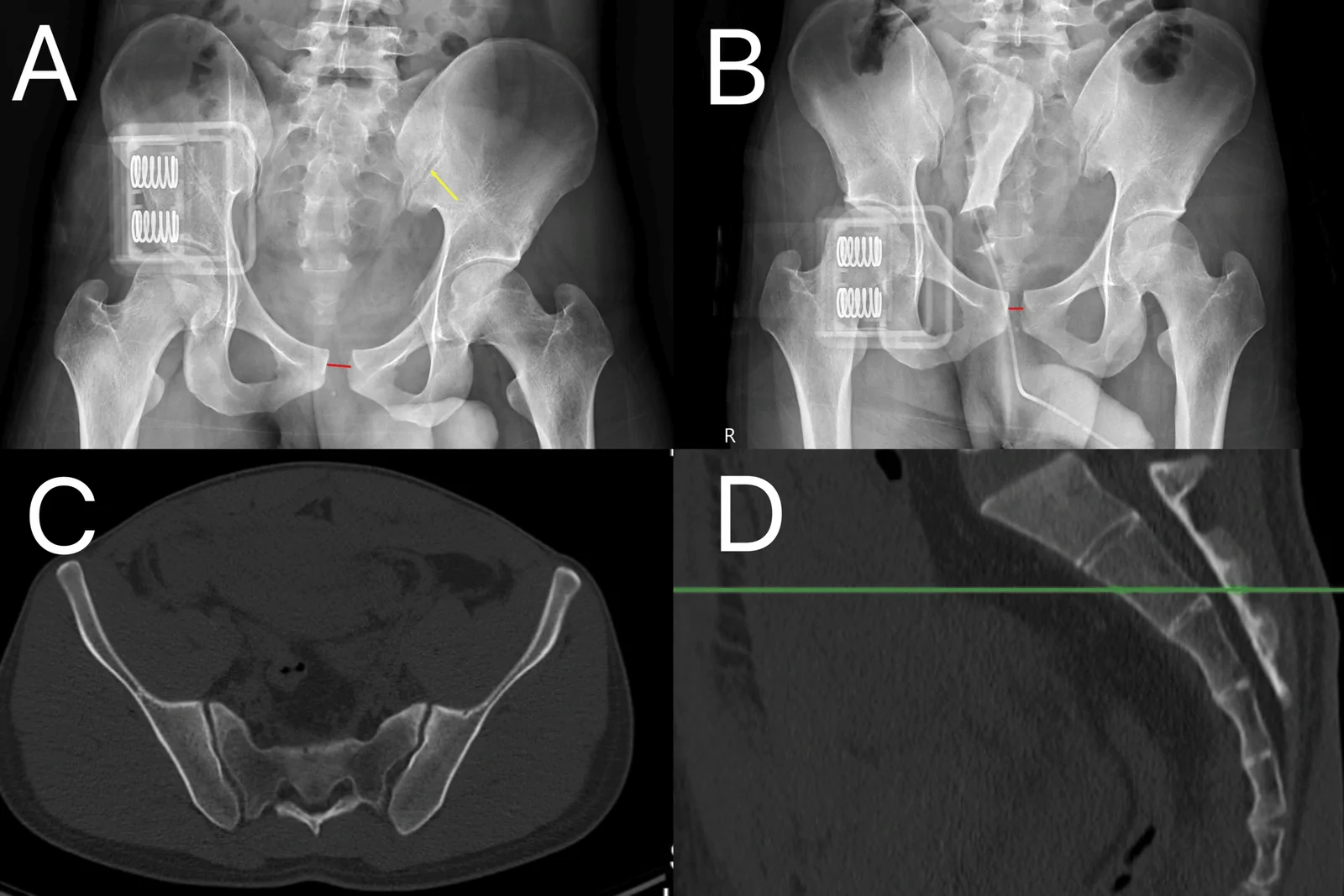
Radiographs on admission A: Initial AP pelvis radiograph showed a 12 mm diastasis of the pubic symphysis (red line) and 2 mm widening of the left sacroiliac joint compared with the contralateral side (yellow arrow). B: AP pelvis radiograph after application and adjustment of a pelvic binder showed reduction of symphyseal diastasis to 7.5 mm (red line). C-D: Computed tomography in axial and sagittal views confirmed an APC type II pelvic ring injury with mild sacroiliac joint widening. AP, anteroposterior; APC, anterior-posterior compression

Associated injuries included a bladder contusion and pelvic hematoma from a left internal iliac artery injury, which were managed nonoperatively.

Initial management

The patient underwent temporary stabilization with anterior pelvic external fixation. Definitive fixation was performed eight days later, consisting of open reduction and internal fixation of the anterior ring via a Pfannenstiel approach using a 3.5 mm reconstruction locking plate and percutaneous posterior fixation with two 7.0 mm cannulated iliosacral screws placed across the left sacroiliac joint under fluoroscopic guidance. Postoperative radiographs demonstrated reduction of symphyseal diastasis to 5 mm (Figure 2).

**Figure 2:**
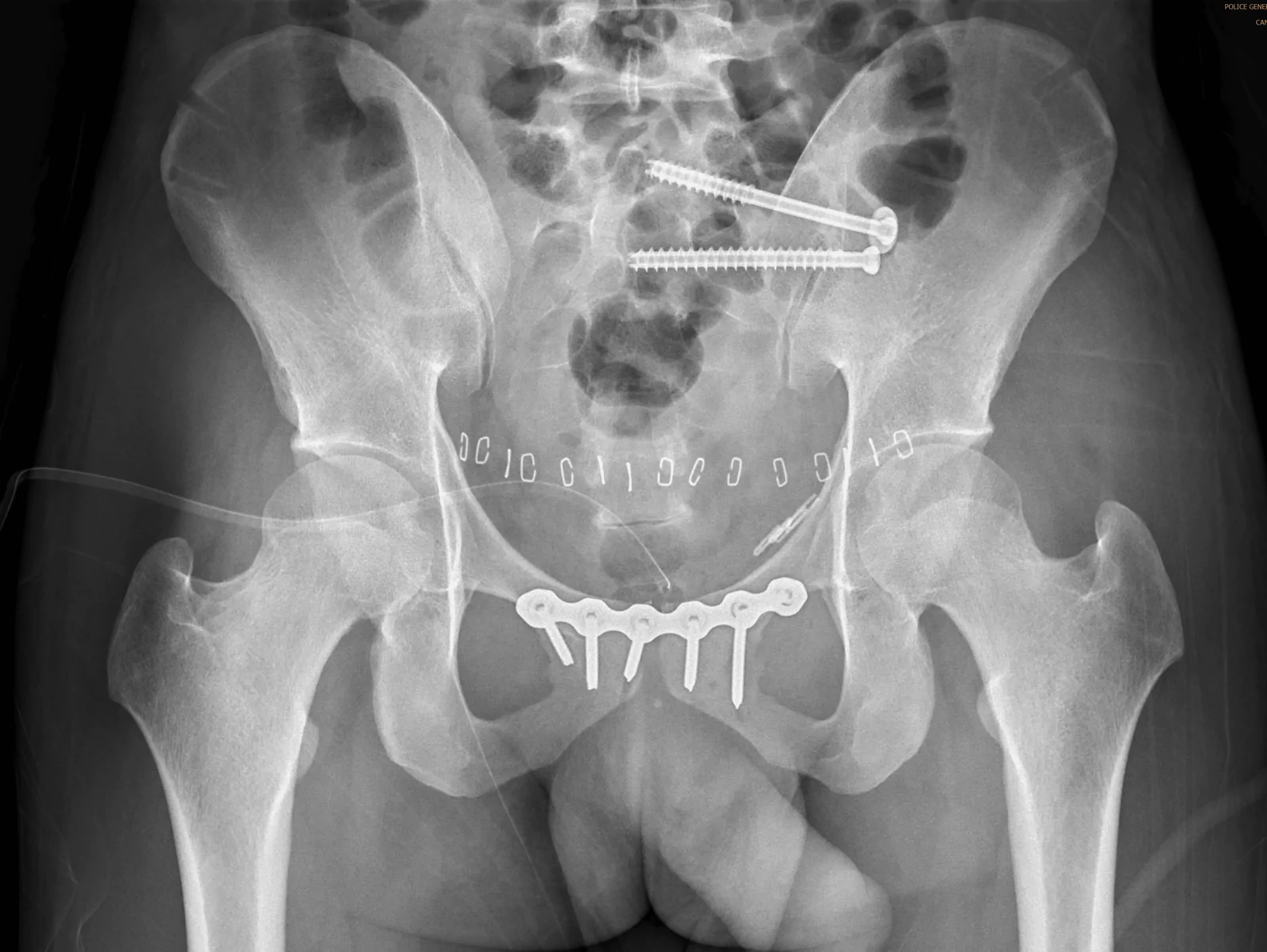
AP pelvis radiograph after definitive fixation Postoperative radiograph showed acceptable reduction of the anterior pubic symphyseal distance to 0.5 cm. AP, anteroposterior

Complication and salvage management

Thirteen days postoperatively, the patient presented with signs of a deep surgical site infection at the anterior incision. Surgical exploration revealed a deep abscess beneath the anterior plate. Removal of the anterior plate and serial surgical debridement, along with placement of antibiotic cement beads, were performed. The posterior iliosacral screws were retained (Figure 3). Intraoperative stress testing after anterior plate removal showed approximately 2 mm of widening of the pubic symphysis.

**Figure 3:**
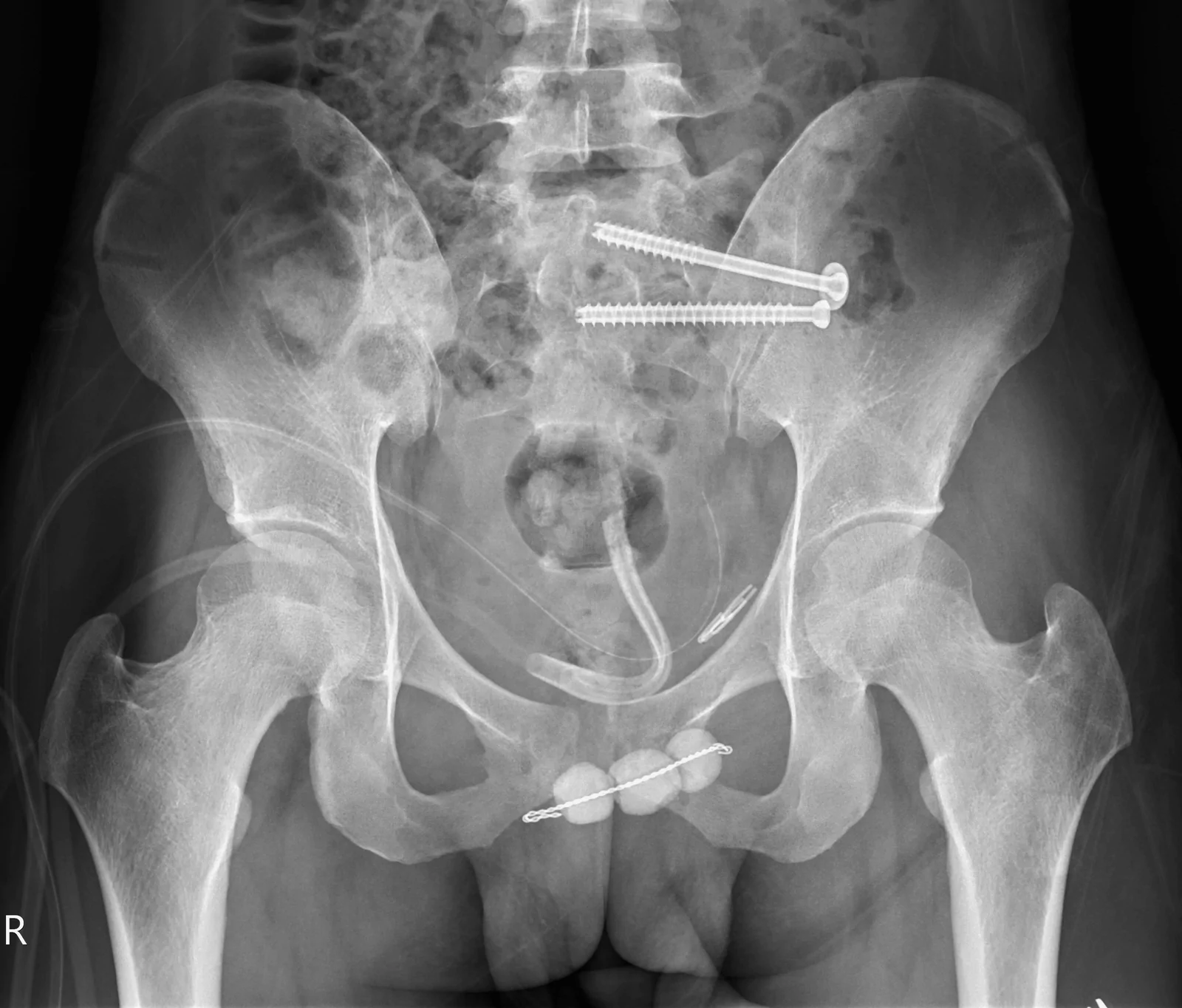
AP pelvis radiograph after removal of the anterior plate Postoperative radiograph showed widening of the anterior pubic symphyseal distance to 0.85 cm. AP, anteroposterior

Follow-up and outcomes

Following implant removal, radiographs demonstrated a mild increase in symphyseal diastasis to 8 mm without progressive displacement. The patient was initially managed with bed rest and non-weight-bearing ambulation. At the fifth week after removal of the anterior plate, he reported no pain at the pubic symphysis or sacroiliac joint. Stable radiographic parameters were observed. At the eighth week, we initiated partial weight-bearing, and at the 11th week, the patient progressed to full weight-bearing ambulation. Serial radiographs demonstrated maintained pelvic alignment with stable symphyseal diastasis of approximately 8 mm (Figure 4).

**Figure 4:**
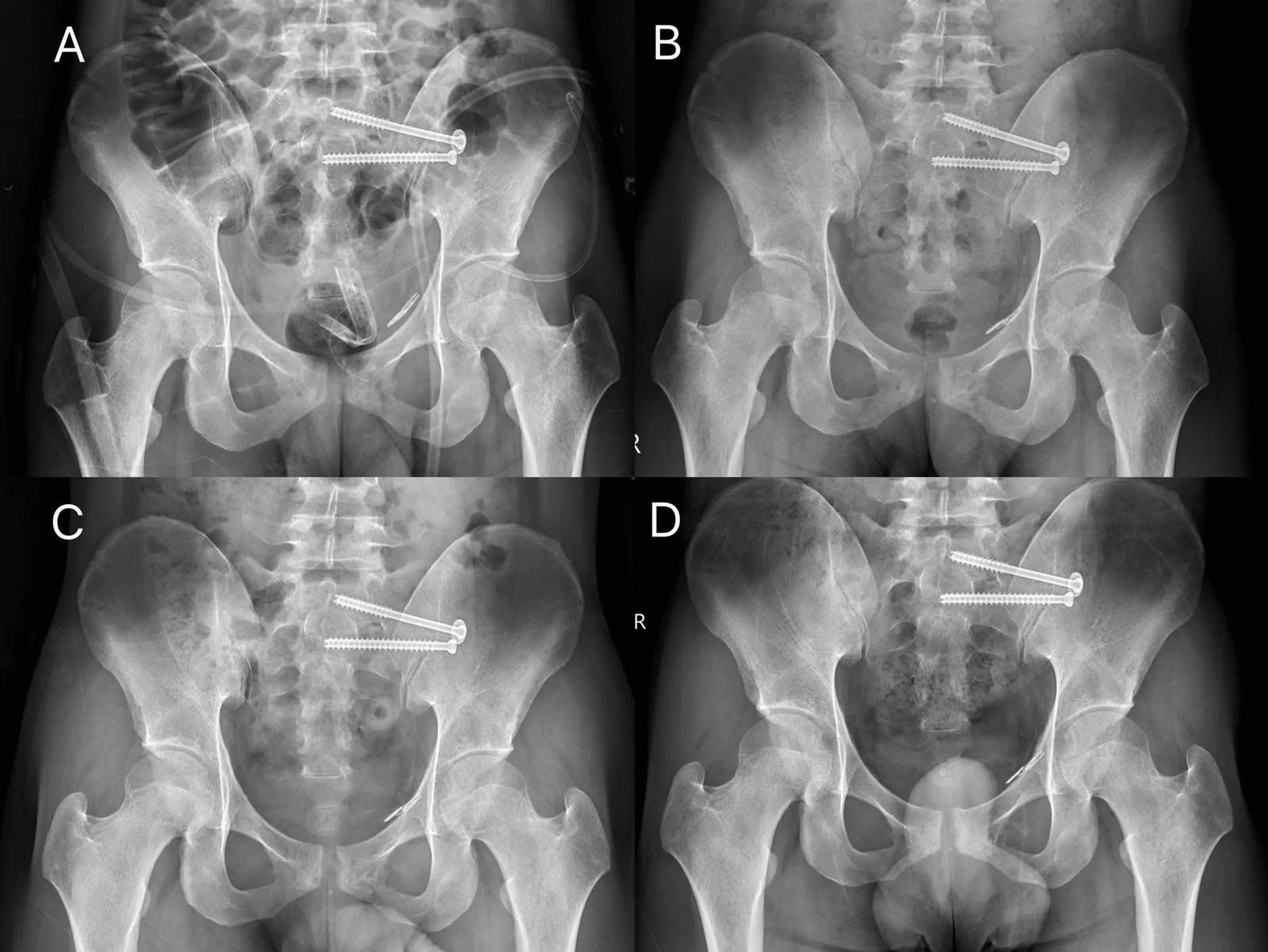
Serial follow-up radiographs showing an unchanged anterior pubic symphyseal distance A: AP pelvis radiograph at the fifth week after removal of the anterior plate showed an anterior pubic symphyseal distance of approximately 0.8 cm. B: AP pelvis radiograph at the eighth week after removal of the anterior plate showed an anterior pubic symphyseal distance of approximately 0.8 cm. C: AP pelvis radiograph at the 11th week after removal of the anterior plate showed an anterior pubic symphyseal distance of approximately 0.8 cm. D: AP pelvis radiograph at six months showed an anterior pubic symphyseal distance of approximately 0.8 cm. AP, anteroposterior

## Discussion

The management of APC type II pelvic injuries remains controversial. While anterior fixation addresses rotational instability, posterior fixation has been shown to enhance overall construct stability and reduce failure rates when used adjunctively [4,6,7].

In this case, anterior fixation was complicated by a deep infection, necessitating implant removal. Despite loss of anterior stabilization, the retained posterior iliosacral screws provided sufficient stability to maintain pelvic alignment and allow progressive mobilization.

Cadaveric biomechanical studies provide insight into the relative contributions of anterior and posterior fixation. Sagi et al. demonstrated that in vertically unstable pelvic injuries, anterior symphyseal plating significantly enhances construct stability and restores normal hemipelvic motion under axial loading. In contrast, the addition of multiple iliosacral screws did not confer a significant biomechanical advantage over a single well-positioned screw. These findings emphasize that while posterior fixation plays a major role in stability, anterior fixation remains critical in restoring physiological load transfer, particularly in resisting abnormal motion patterns [11].

Conversely, van Zwienen et al. reported that in unstable pelvic ring injuries, the use of multiple sacroiliac screws increases rotational stiffness and improves load-to-failure characteristics compared to a single-screw construct [12]. Recent studies have explored posterior-only fixation strategies in more unstable pelvic injuries, suggesting comparable outcomes in carefully selected patients [13,14].

Taken together, these studies suggest that posterior fixation contributes substantially to overall construct strength, that anterior fixation plays a key role in controlling rotational instability and restoring normal biomechanics, and that the effectiveness of posterior fixation depends on construct configuration.

In APC type II injuries, where posterior ligamentous structures are only partially disrupted, residual intrinsic stability may allow posterior fixation to maintain alignment once reduction has been achieved. In the present case, the combination of adequate initial reduction, preserved posterior fixation, and controlled postoperative mobilization likely contributed to maintenance of stability following anterior implant removal.

Importantly, these findings should not be interpreted as supporting posterior-only fixation as a primary treatment strategy in APC type II injuries. Rather, this case demonstrates that in a reduced pelvis with intact posterior fixation, isolated posterior stabilization may be sufficient to maintain alignment under specific salvage conditions.

This distinction is clinically relevant in scenarios such as infection, soft tissue compromise, or urologic injury, where anterior fixation may not be feasible or must be removed.

Limitations

This report describes a single case, limiting generalizability. Functional outcomes were assessed clinically without standardized scoring systems.

## Conclusions

This case demonstrates that isolated posterior fixation may provide sufficient pelvic ring stability to permit healing and progressive functional recovery in a carefully selected patient with an APC type II pelvic ring injury following unplanned early removal of anterior fixation due to a deep surgical site infection. Despite loss of anterior fixation, only minimal residual symphyseal widening was observed on intraoperative stress examination, and pelvic alignment remained stable during subsequent follow-up with a gradual progression from protected to full weight-bearing ambulation.

These findings suggest that, when adequate posterior stabilization has been achieved and residual anterior instability is limited, retention of posterior fixation without immediate anterior re-fixation may represent a reasonable salvage strategy in situations where anterior hardware cannot be safely retained. However, this approach should not be interpreted as supporting routine posterior-only fixation as the primary treatment for APC type II injuries. Careful assessment of pelvic stability, fracture pattern, quality of posterior fixation, and serial radiographic findings remains essential when considering this strategy. Further biomechanical and prospective clinical studies with larger patient cohorts are required to determine the appropriate indications, safety, and long-term functional outcomes of posterior-only fixation in this setting.
